# LNA probes substantially improve the detection of bacterial endosymbionts in whole mount of insects by fluorescent *in-situ* hybridization

**DOI:** 10.1186/1471-2180-12-81

**Published:** 2012-05-24

**Authors:** Natarajan Gayatri Priya, Neeti Pandey, Raman Rajagopal

**Affiliations:** 1 Gut Biology Lab, Department of Zoology, University of Delhi, Room No 117, Delhi, 110007, India

## Abstract

**Background:**

Detection of unculturable bacteria and their localization in the host, by fluorescent *in-situ* hybridization (FISH), is a powerful technique in the study of host-bacteria interaction. FISH probes are designed to target the 16 s rRNA region of the bacteria to be detected. LNA probes have recently been used in FISH studies and proven to be more efficient. To date no report has employed LNA probes for FISH detection of bacterial endosymbiont in the whole mount tissues. Further, though speculated, bacteriocytes have not been reported from males of *Bemisia tabaci*.

**Results:**

In this study, we compared the efficiency in detecting bacteria by fluorescent DNA oligonucleotides versus modified probes containing Locked Nucleic Acid (LNA) substitution in their structure. We used the insect *Bemisia tabaci* as the experimental material since it carried simultaneous infection by two bacteria: one a primary endosymbiont, *Portiera* (and present in more numbers) while the other a secondary endosymbiont *Arsenophonus* (and present in less numbers). Thus a variation in the abundance of bacteria was expected. While detecting both the bacteria, we found a significant increase in the signal whenever LNA probes were used. However, the difference was more pronounced in detecting the secondary endosymbiont, wherein DNA probes gave weak signals when compared to LNA probes. Also, signal to noise ratio for LNA probes was higher than DNA probes. We found that LNA considerably improved sensitivity of FISH, as compared to the commonly used DNA oligonucleotide probe.

**Conclusion:**

By employing LNA probes we could detect endosymbiotic bacteria in males, which have never been reported previously. We were able to detect bacteriocytes containing *Portiera* and *Arsenophonus* in the males of *B. tabaci*. Thus, employing LNA probes at optimized conditions will help to significantly improve detection of bacteria at the lowest concentration and may give a comprehensible depiction about their specific distribution within samples.

## Background

Microscopic detection and localization of a specific DNA or RNA segment within single cells or a histological section has been made possible with the advent of the *In-Situ* Hybridization (ISH) technique. This technique relies principally on formation of Watson-Crick base pairing between the gene of interest and the applied complementary sequence to which the reporter molecule is attached [1]. Fluorescent *in-situ* hybridization (FISH) is an extension of this technique in which a fluorophore tagged to the probe, acts as the reporter molecule. FISH is a widely used technique in clinical studies relating to diagnosis, prognosis and sometimes, even remission of diseases like cancer [2,3]. In microbiology, studies pertaining to microbial ecology employ FISH in detection and identification of unculturable microbes in clinical and environmental samples as well as whole mount tissues [4,5]. Some studies have employed FISH for revealing the distribution pattern of two very closely related (<3% difference in nucleotide sequence) species of marine cyanobacteria [6]. DNA oligonucleotide probes are most commonly used when compared to ssDNA, dsDNA or RNA probes due to features like: stability, ease of availability and cost effectiveness.

A modification in the structure of nucleotides with methylene bridge to connect 2’ oxygen and 4’ carbon of the ribose ring, gives rise to Locked Nucleic Acid (LNA) [7]. The extra bridge in an LNA structure makes the ribose moiety inaccessible, thereby locking the structure to high binding affinity conformation [8,9]. Such LNA nucleotides can be mixed with DNA or RNA residues during synthesis of oligonucleotide to enhance the hybridization specificity, sensitivity and duplex stability [8,10]. When compared to DNA only oligonucleotide probes, it is seen that LNA modified DNA oligonucleotide probes (hereinafter called LNA probes) are 10 fold more sensitive when applied in techniques like northern analysis [11]. LNA probes have also been successfully used for FISH to identify individual *E. coli* cells [12]. They have been used for temporal and spatial detection of miRNAs or mRNA by whole mount ISH and in tissue sections [13-16]. Some studies have used LNA in clinical studies for detection and differentiation between two fungal pathogens in tissue sections [17-19]. There are many reports that have identified and localized bacteria by targeting 16 S rRNA gene in whole mount or microtome section samples but till date there has been no report wherein LNA probes have been employed for bacterial detection by FISH in whole mount or microtome section of biological samples.

The insect *Bemisia tabaci*, commonly known as whitefly, is an agricultural pest with a wide host range. *B. tabaci* is a vector of a group of plant viruses known as *Geminiviruses* which significantly damage the host plant. Recent studies have linked the transmission of *Tomato Yellow Leaf Curl virus* (TYLCV), to the GroEL protein of a secondary endosymbiont of *B. tabaci*[20]. Therefore, an extensive study of the type and nature of spread of *B. tabaci* endosymbionts is primary to understanding their functional role within the host insect. Two types of endosymbionts are reported to be present within the *B. tabaci*, namely the primary endosymbiont and the secondary endosymbiont [21]. Whiteflies are one of the rare cases in which co-infection, of primary and secondary symbionts, occurs in the same cell [22]. Therefore, in this study we have compared the efficiency of both DNA only and LNA modified DNA probes in the detection and localization of a primary endosymbiont that is present in abundance, as well as a secondary endosymbiont that is less abundant in nature.

## Methods

We collected adult *Bemisia tabaci* from cotton leaves from fields of Indian Agricultural Research Institute (Pusa, New Delhi, India), washed them with ethanol and water, and stored in acetone at −20°C till further processing. The specimens were processed using standardized method of Gottlieb et al [21] for whitefly with slight modifications. *B. tabaci* specimens were stored overnight in Carnoy’s fixative (chloroform: ethanol: glacial acetic acid, 6:3:1) and decolorized with 6% H_2_O_2_ in ethanol for 24 hrs. *Portiera* and *Arsenophonus* detection was performed using FAM labeled probe bearing 5’ TGTCAGTGTCAGCCCAGAAG 3’ sequence and TYE-665 probe bearing of 5’ TCATGACCACAACCTCCAAA 3’ sequence respectively [20]. The DNA probe and modified LNA were supplied by Exiqon A/S [the exact positions of the LNA modifications of *Portiera* (batch no. 5032716, containing 5 LNA) and *Arsenophonus* (batch no. 503274, containing 6 LNA), are not known to us]. The decolorized insects were hybridized at 40°C, with the DNA and LNA probes, in hybridization buffer (20 mM Tris-Cl [pH 8.0], 0.9 M NaCl, 0.01% sodium dodecyl sulfate) containing increasing amount of formamide (0%-80%). Probe concentrations of 0.6 pmoles for *Portiera* and 1.0 pmoles for *Arsenophonus* were kept identical for LNA and DNA. After the overnight incubation, the samples were thoroughly washed in a washing buffer (0.3 M NaCl, 0.03 M sodium citrate, 0.01% sodium dodecyl sulfate) for 5 minutes and mounted using Vectashield (Vector Labs). Each of the endosymbiont was detected at 9 different formamide concentrations (0% - 80%) separately, with DNA as well as LNA probes. Replicates consisted of 10 insects for each condition. Specificity of detection was confirmed using no probe staining and RNase- digested specimen staining. All the images were acquired at fixed camera and microscope settings for DNA and LNA with Nikon A1 confocal microscope. The fluorescence intensities were quantified by NIS elements (V 3.21.02) image analysis software (Nikon).

## Results and discussion

The primary endosymbiont of *Bemisia tabaci* is *Portiera*[23]. This symbiont is housed exclusively in specialized structures called bacteriocytes [24]. Since this insect cannot survive without its obligate primary endosymbiont, these symbionts are present in higher proportion or abundance than other secondary endosymbionts. FISH studies pertaining to localization of *Portiera* using confocal microscope has been described earlier [21]. *Arsenophonus* is a secondary endosymbiont whose exact role is yet to be ascertained and whose population within the insect is lower than that of *Portiera*. Location of *Arsenophonus* is reported to be in the same cell as *Portiera* i.e. the bacteriocytes [22].

### Comparing LNA and DNA probes to detect *Portiera* the primary bacterial endosymbiont of *Bemisia tabaci*

**Figure 1:**
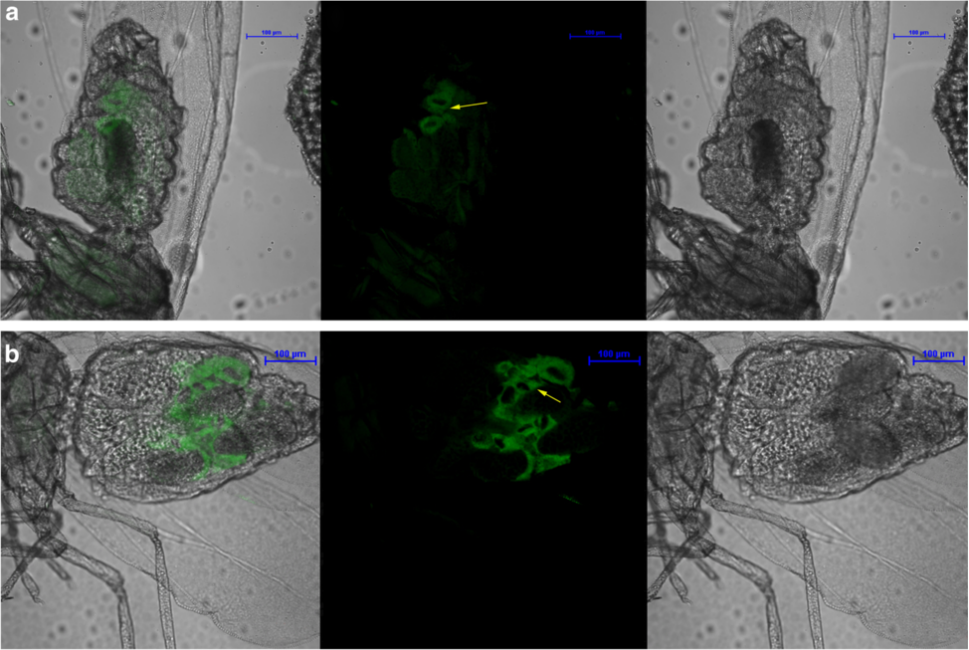
** FISH staining of *Portiera *16 S rRNA in whole mount of whitefly *Bemisia tabaci. *** FAM labeled oligonucleotide DNA probe and modified LNA probes were used to detect *Portiera* in *B*. *tabaci*. (**A**.**b**) DNA probe stains for *Portiera* in the bacteriocytes (**B**.**b**) at the same concentration (0.6 pmoles) LNA probe shows higher signal and lower background while staining for *Portiera*. Arrows indicate the bacteriocytes. The images have been taken at best formamide concentration for *Portiera* DNA (40%) and LNA (60%) probes separately. Both DNA and LNA panels also show merged and DIC images (as a and c respectively). All the images were acquired at fixed camera and microscope settings with Nikon A1 confocal microscope.

**Figure 2:**
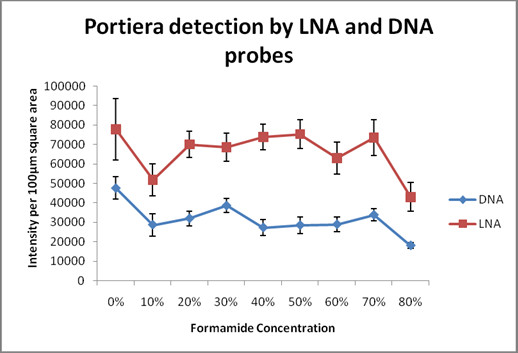
** Comparison between LNA and DNA probes while detecting the more abundant endosymbiont (*Portiera *).** This graph depicts signal intensity profiles of LNA and DNA probes as a function of formamide concentration after background subtraction. At the same concentration, the intensity profile of LNA probe is significantly higher than the DNA probe while detecting *Portiera*, an endosymbiont of high abundance. All the images were acquired at fixed camera and microscope settings for DNA and LNA with Nikon A1 confocal microscope. Fluorescence intensities were quantified by NIS elements (V 3.21.02) image analysis software (Nikon).

While detecting *Portiera* we found LNA to be more sensitive than DNA oligonucleotide probes (Figure 1). At 0% formamide concentration, we observed very high DNA and LNA signals, but these samples also showed very high background noise [12] and hence we excluded it from analysis. DNA probe had highest intensity values (~30,000) at 30% formamide concentration (Figure 2). All intensity measurements were done after background correction. Previous studies [25] with DNA probes detecting *Portiera* have used 30% formamide concentration for their FISH experiments, which is in agreement to our result obtained from DNA probe. The LNA signals (~70,000) peaked at 50% formamide concentration. The signal intensities of both DNA and LNA probes varied only to some extent with increasing formamide concentrations. Negative controls did not show any signal for *Portiera* (Additional file 1: Figure S1 & Additional file 2: Figure S2). Overall, it was clearly evident that in most of the formamide concentrations, LNA probes had signal intensity nearly 2 times (and sometimes even more) as high as its DNA counterpart when detecting *Portiera*. 

**Figure 3:**
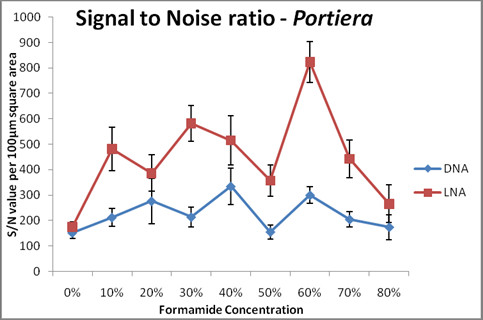
** Signal to noise ratio of LNA and DNA probes while detecting the more abundant endosymbiont (*Portiera*).** This graph depicts the signal to noise ratio, per 100 μm square area and plotted against increasing formamide concentration. No background correction was performed here. The value was calculated by dividing signal with the background of the same image and thus it gives a good idea about the binding efficiency of the probe. Here, LNA probe has a high signal to noise ratio at 60% formamide concentration followed by 30% formamide concentration, when compared to DNA probe. The signal of LNA probe is always high than the DNA probe at all formamide concentrations. *Portiera* was detected at 9 different formamide concentrations (0%-80%), both by DNA as well as the LNA probes. Fluorescence intensities were quantified by NIS elements (V 3.21.02) image analysis software (Nikon).

The Signal to Noise (S/N) value is an indicator of sensitivity of the probe since it is a measure of both the signal and the background. For this purpose no background correction was done, so that along with the actual signals of *Portiera*, the background noise of DNA and LNA could also be calculated for the same samples for 100 μm^2^ area respectively. S/N ratio value was obtained by dividing signal intensity with the background noise. Figure 3, where S/N ratio is plotted against increasing formamide concentration compares the two probes. The LNA probe had nearly twice as much S/N values as DNA probe, while detecting *Portiera*. The highest S/N value (823) was obtained with LNA probe at 60% formamide concentration. Use of high formamide concentration for LNA probes in order to reduce the background noise, has been previously performed when detecting lactic acid bacteria [26]. In DNA probe the highest S/N value (334) was at 40% formamide concentration. It was evident from the graph that the LNA probe has higher signal and lower noise ratio than DNA at all formamide concentrations. At 0% formamide concentration even though the main signal is high, an equally high background noise reduces the S/N ratio value in both DNA and LNA probes. In agreement to previous studies [12], we find that high sensitivity and stringency can be obtained by using LNA probes at high formamide concentrations while performing FISH in insect whole mounts. 

### Comparing LNA and DNA probes to detect *Arsenophonus* the secondary bacterial endosymbiont of *Bemisia tabaci*

**Figure 4:**
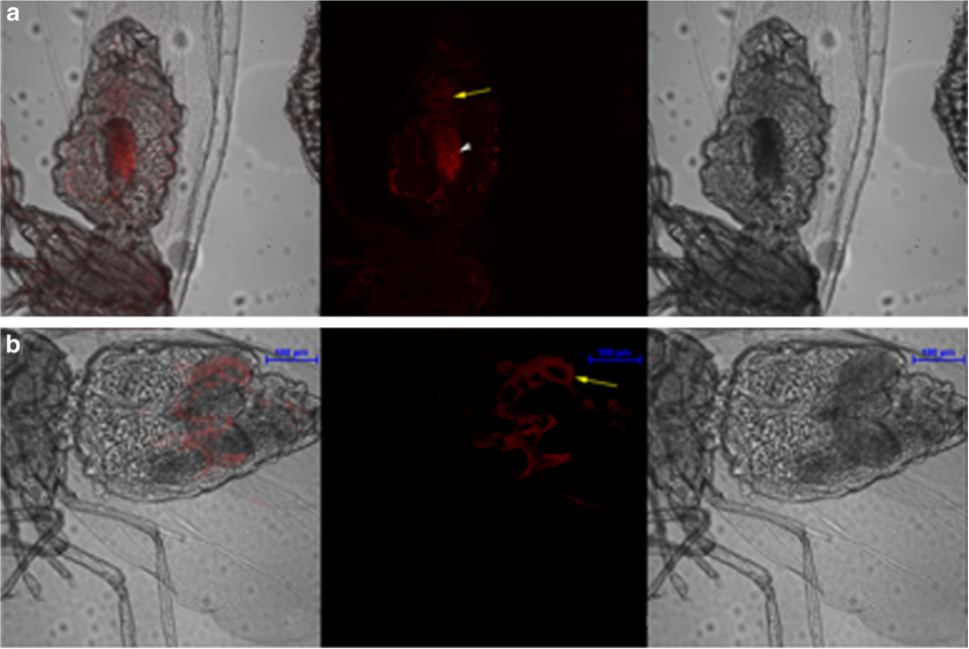
** FISH staining of *Arsenophonus *16 S rRNA in whole mount of whitefly *Bemisia tabaci *.** (**A**.**b**) DNA probe stains *Arsenophonus* in the bacteriocytes; (**B**.**b**) at the same concentration (1.0 pmoles) LNA probe shows higher signal and a low background while staining for *Arsenophonus*. Arrows indicate the bacteriocytes. White arrowhead indicates the non-specific background in DNA samples. The images have been taken at best formamide concentration for *Arsenophonus* DNA (30%) and LNA (70%) probes separately. Both DNA and LNA panels also show merged and DIC images (as **a** and **c** respectively).

FISH detection of *Arsneophonus* 16 S rRNA was performed keeping all the conditions, but the laser settings, similar for DNA and LNA probes (Figure 4). This was done because, at laser settings where we could pick up signals of LNA, DNA did not show any detection signal (Additional file 3: Figure S3A and S 3B). Also, when the laser power, HV and offset were increased with regard to DNA probe, LNA probe increased multifold in signal intensity and background (Additional file 3: Figure S3C). The laser settings were then lowered for LNA probe to such an extent that even the lowest signal produced by LNA was detectable. Different probe concentrations were also tested for DNA and LNA in order for detecting *Arsenophonus* where 1 pmoles concentration showed good results*.* At lower probe concentration (0.6pmoles) that was used for detection of *Portiera*, DNA failed to produce any signal for *Arsenophonus,* even though non-specific background signals could still be detected (Additional file 4: Figure S4A). LNA probe produced low intensity signals at the same concentration (Additional file 4: Figure S4B).

**Figure 5:**
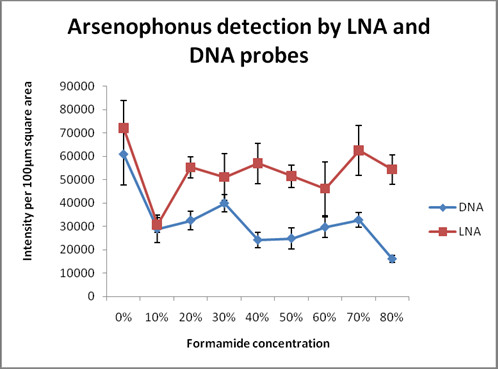
** Comparison between LNA and DNA probes for detecting endosymbiont of lower abundance (*Arsenophonus*).** All specimens were processed using the procedure described for *Portiera*. However, the probe concentration used for *Arsenophonus* was 1.0 pmoles and kept identical for LNA and DNA. This graph depicts the signal intensity profiles of LNA and DNA probes as a function of formamide concentration after background subtraction. At the same concentration, the intensity profile of LNA probe is significantly higher than the DNA probe while detecting *Arsenophonus*, an endosymbiont of low abundance. Fluorescence intensities were quantified by NIS elements (V 3.21.02) image analysis software (Nikon).

We found that LNA probes produced very high signals when compared to the DNA probes (Figure 4) while detecting *Arsenophonus*. We performed all the intensity measurements only after background correction. The LNA probe had highest intensity values (>60,000) at 70% formamide concentration while the lowest (30,000) at 10%. DNA probe had highest intensity at 30% formamide concentration (39,000) and lowest at (16,000) 80% formamide concentration. At 10% formamide concentration, LNA signal was nearly as low as the DNA signal (Figure 5). The DNA probe gave an intensity which was similar to that of LNA probe at 0% formamide concentration. Similar to the earlier case of *Portiera*, 0% formamide gave high signal intensity as well as very high background noise. Therefore we did not consider it as an ideal concentration to detect the difference between the probes. It was seen that DNA probe produced good signal only at very low formamide concentration unlike LNA probe. Negative controls did not show any signal for *Arsenophonus* (Additional file 1: Figure S1 & Additional file 2: Figure S2). Since high formamide concentration produces high stringency, false positive signals get negated while using LNA probes.

**Figure 6:**
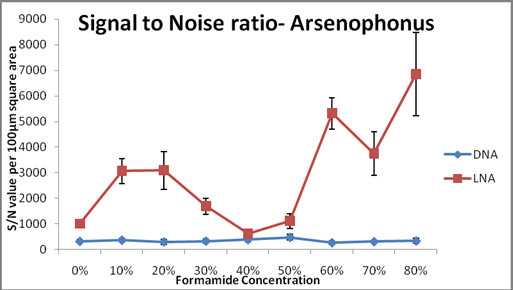
** Signal to noise ratio of LNA and DNA probes while detecting the less abundant endosymbiont (*Arsenophonus*).** The graph depicts the signal to noise ratio, per 100 μm square area and plotted against increasing formamide concentration. No background correction was performed here. S/N value was calculated by dividing signal with the background of the same image and thus it gives a good idea about the binding efficiency of the probe. LNA has a high signal to noise ratio at all formamide concentrations, when compared to DNA probe. The high signal and low background of LNA probes was observed even when the laser settings were lower than that of DNA probes. *Arsenophonus* was detected at 9 different formamide concentrations (0%-80%), both by DNA as well as the LNA probes. Replicates consisted of 10 insect samples for each condition. Fluorescence intensities were quantified by NIS elements (V 3.21.02) image analysis software (Nikon).

We then compared the sensitivity profiles of both the probes based on Signal to Noise (S/N) ratio. For S/N ratio calculation, no background correction was performed, so that the background noise and actual signals could be recorded per 100 μm^2^ area for both DNA and LNA probes in *Arsenophonus* samples. We calculated the S/N ratio and found that LNA values were significantly higher than the DNA values (Figure 6). At 80% formamide concentration, the highest S/N value of LNA probe (6852) was 20 times the S/N values of DNA probe (331) at the same concentration. 60% formamide concentration was equally effective for LNA probes. The S/N ratio value for LNA probe (602) dipped lower at 40% formamide concentration, which was still more than the S/N value of DNA probe (381) at the same formamide concentration. The DNA probe had highest S/N value (472) at 50% formamide concentration and lowest value (265) at 60% formamide concentration. It needs to be noted that the statistically important difference between LNA probe and DNA probe prevailed in spite of the low laser settings for former’s detection. LNA probe detected *Arsenophonus* as sensitively as *Portiera*, irrespective of the endosymbiont’s abundance, thereby proving its high efficiency compared to DNA probe.

The results presented here show that apart from many other applications reported so far [11-19], modified LNA probes are more effective for detecting bacteria in whole mounts of insect tissue than the conventional DNA oligonucleotide probes. This is because LNA probes are stable against 3'-exonucleolytic degradation and possess excellent aqueous solubility [27]. Additionally, the charged phosphate backbone of LNA oligonucleotides allows them to be transfected and taken up by cells just like DNA, thus finding use in many biological applications [7,15,26]. LNA modification of oligonucleotides reduces flexibility and results in more stable duplex structures [8]. The integration of 2–4 LNAs with oligonucleotides increases their binding to 16 S ribosomal RNA by up to 22-fold [12]. The improvement in detecting the endosymbionts of interest by LNA probes, when compared to DNA counterpart, is due to their increased thermodynamic stability and improved discrimination between perfectly matched and mismatched target nucleic acids [27]. It can be suggested that the features like higher melting temperature, better tissue penetrability and target accessibility [28] are the reasons why LNA outperforms DNA at nearly all formamide concentrations.

### Detection of bacteriocytes in male *B. Tabaci*

**Figure 7:**
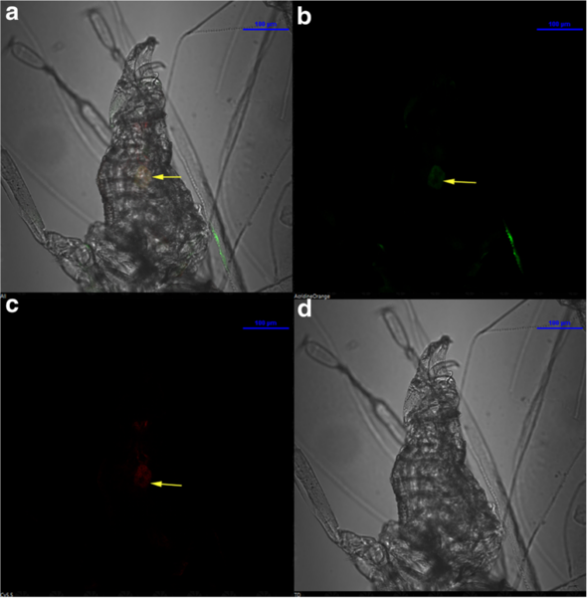
** FISH staining of bacteriocyte in *Bemisia tabaci *male.** The LNA probe details remain similar to those described in Figure 1 and 4. (**A**.**b** &**A**.**c**) LNA probe stains *Portiera* and *Arsenophonus* in the bacteriocytes of adult male; Arrows in yellow indicate the bacteriocytes. The panel also shows merged and DIC images (as **A**.**a** and **A**.**d** respectively).

Having concluded that LNA probes are better, we then tried to unravel more information than already reported regarding the distribution of endosymbionts using these probes. It has been reported that in *B. tabaci*, *Portiera* is present exclusively in the bacteriocytes and more so, easily detectable only in adult females [21]. Even though males are considered evolutionarily dead, due to the fact that they do not transmit symbionts to the offspring, studies in other insects like carpenter ants indicate that males do inherit endosymbionts for survival during their lifetime [29]. Earlier reports about bacterial symbiont localization have never reported any localization within males of *B. tabaci*[22,25]. Since from our previous results, 60% formamide concentration for both *Portiera* and *Arsenophonus* produced high signal and low background, we considered it optimum for our investigation with LNA probes. We have detected for the first time, using LNA probes, not only *Portiera* but *Arsenophonus* signals as well, within the bacteriocytes of adult males (Figure 7). These endosymbionts, however, could not be detected when we used DNA oligonucleotide probes for staining. 

## Conclusion

Further studies using LNA probes for whole mount FISH can give us a better idea about the spread of endosymbionts and the various niches occupied by them within a tissue sample. In *B. tabaci* the use of LNA probes for detection of other endosymbionts will provide better understanding about the fly. Use of LNA can also be extended to the level of visualizing the existing interaction between the virus and the endosymbionts.

## Authors’ contributions

NGP and NP collected the samples. NGP performed the experiments, analyzed the data and wrote the paper. RR edited the paper and designed the research. All authors read and approved the final manuscript.

## Supplementary Material

Additional file 1 ** Figure S1.** FISH staining of *Portiera* and *Arsenophonus* in whole mount of whitefly *B. tabaci* in RNase digested insect sample. No signal is detected for either *Portiera* (A.b) or *Arsenophonus* (A.c) when using LNA probes at similar conditions as in Figures 1 and 4. a and d panels show the merged and DIC images. (TIFF 5425 kb) Click here for file

Additional file 2 ** Figure S2.** Negative control without any probe. No signal was detected in the negative control. a and d panels show the merged and DIC images. (TIFF 4571 kb) Click here for file

Additional file 3 ** Figure S3.** FISH staining of *Arsenophonus* in whole mount of whitefly *B. tabaci* at different laser settings. At low laser settings, the signal produced by DNA probe for *Arsenophonus* was not detectable (A.b). While LNA probe at the same settings could easily detect bacteria, giving good signal and minimum or no background (B.b). But when laser power was increased such that DNA probe signal could be detected, the LNA probe showed very high signal sensitivity and background (C.b). a and c panels show the merged and DIC images. (TIFF 295 kb) Click here for file

Additional file 4 ** Figure S4.** FISH staining of *Arsenophonus* in whole mount of whitefly *B. tabaci* at low probe concentration. Following the protocol as described, at lower probe concentration (0.6 pmoles) we could not detect *Arsenophonus* using DNA probe (A.b). LNA probe detects *Arsenophonus* at the same probe concentration (B.b). a and c panels show the merged and DIC images of the respective probes. Click here for file
