# Supplementary figures and images for: LNA probes substantially improve the detection of bacterial endosymbionts in whole mount of insects by fluorescent in-situ hybridization
Source: BMC Microbiol. 2012 May 24;12:81. doi: 10.1186/1471-2180-12-81 (PMC3536699; doi:10.1186/1471-2180-12-81)

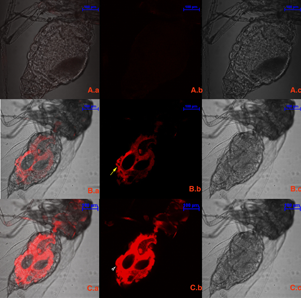

Supplement: Additional file 1 — Figure S1. FISH staining of Portiera and Arsenophonus in whole mount of whitefly B. tabaci in RNase digested insect sample. No signal is detected for either Portiera (A.b) or Arsenophonus (A.c) when using LNA probes at similar conditions as in Figures 1 and 4. a and d panels show the merged and DIC images. (TIFF 5425 kb) [file 1471-2180-12-81-S1.tiff]

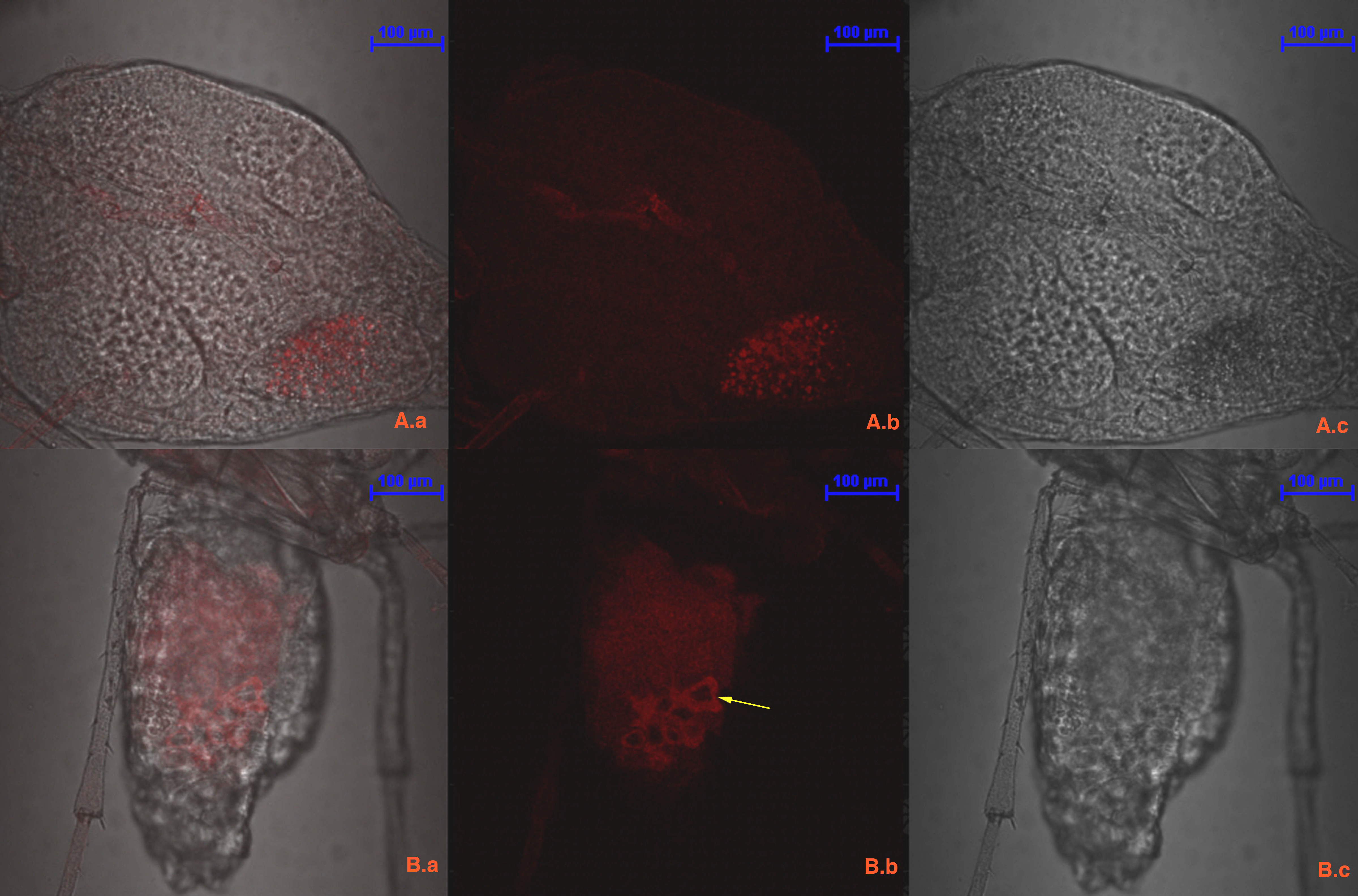

Supplement: Additional file 2 — Figure S2. Negative control without any probe. No signal was detected in the negative control. a and d panels show the merged and DIC images. (TIFF 4571 kb) [file 1471-2180-12-81-S2.tiff]

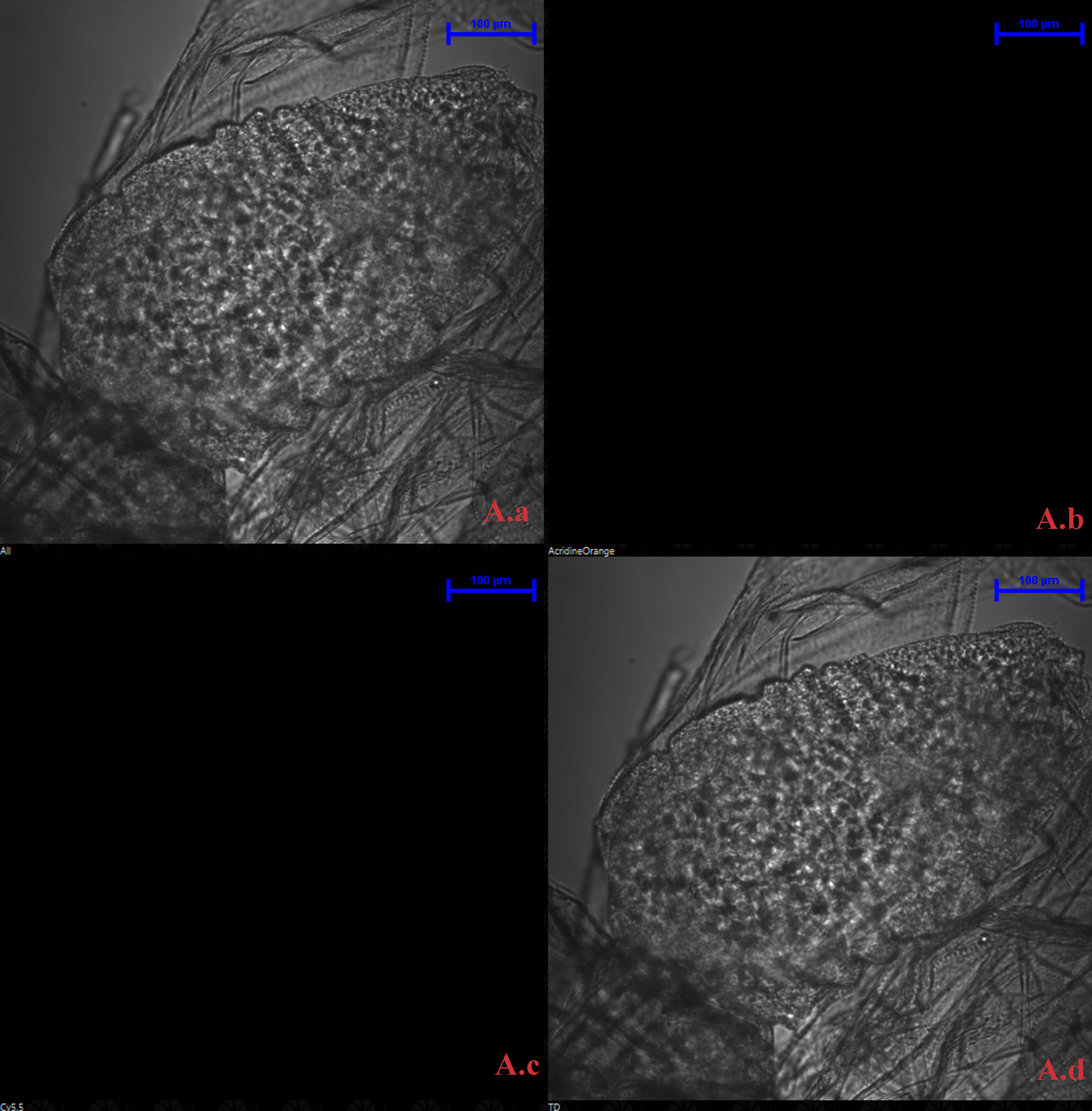

Supplement: Additional file 3 — Figure S3. FISH staining of Arsenophonus in whole mount of whitefly B. tabaci at different laser settings. At low laser settings, the signal produced by DNA probe for Arsenophonus was not detectable (A.b). While LNA probe at the same settings could easily detect bacteria, giving good signal and minimum or no background (B.b). But when laser power was increased such that DNA probe signal could be detected, the LNA probe showed very high signal sensitivity and background (C.b). a and c panels show the merged and DIC images. (TIFF 295 kb) [file 1471-2180-12-81-S3.tiff]

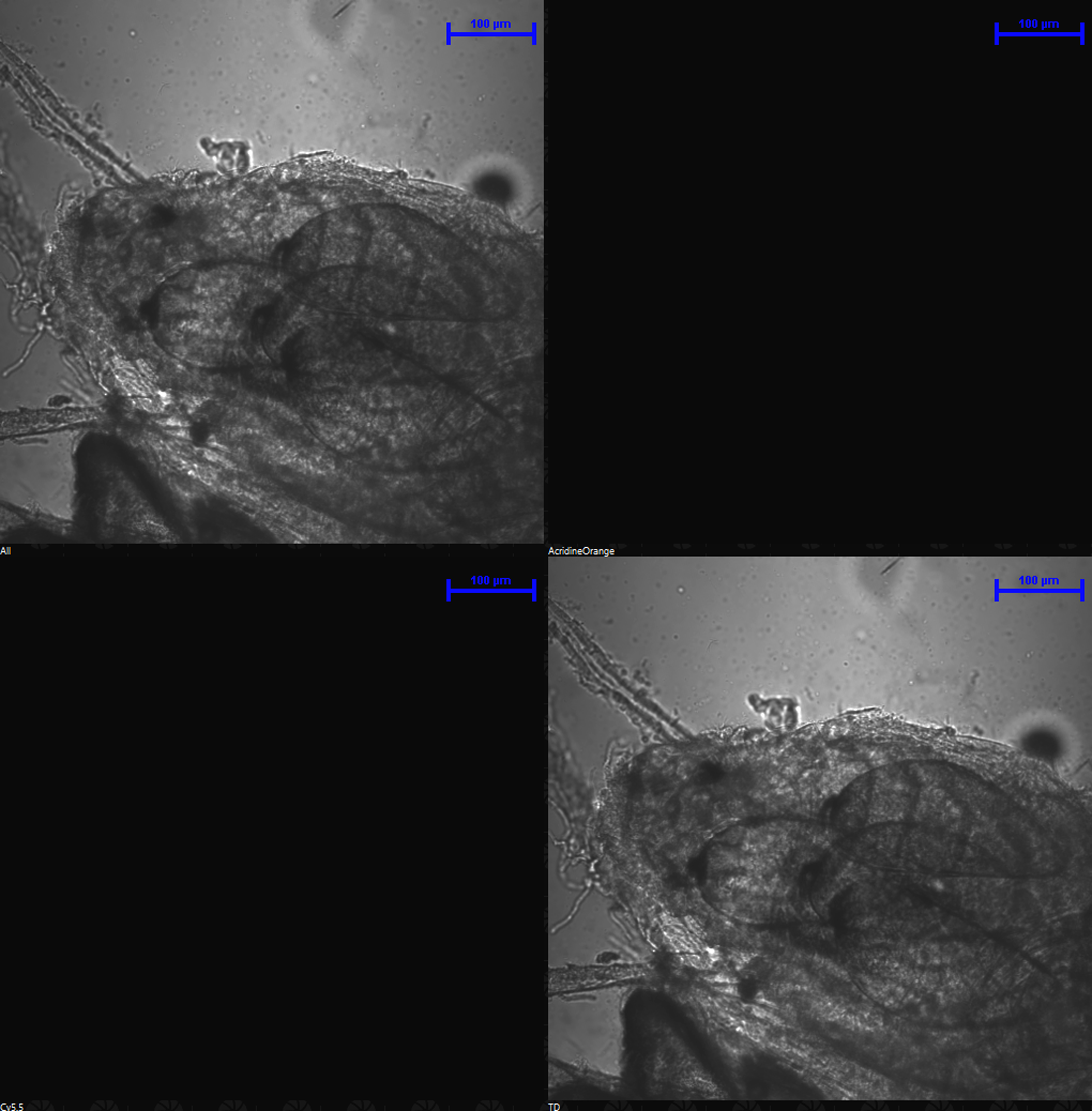

Supplement: Additional file 4 — Figure S4. FISH staining of Arsenophonus in whole mount of whitefly B. tabaci at low probe concentration. Following the protocol as described, at lower probe concentration (0.6 pmoles) we could not detect Arsenophonus using DNA probe (A.b). LNA probe detects Arsenophonus at the same probe concentration (B.b). a and c panels show the merged and DIC images of the respective probes. [file 1471-2180-12-81-S4.tiff]
